# Occurrence of *Leishmania infantum* in Wild Mammals Admitted to Recovery Centers in Spain

**DOI:** 10.3390/pathogens12081048

**Published:** 2023-08-16

**Authors:** Iris Azami-Conesa, Paula Pérez-Moreno, Pablo Matas Méndez, Jose Sansano-Maestre, Fernando González, Marta Mateo Barrientos, María Teresa Gómez-Muñoz

**Affiliations:** 1 Department of Animal Health, Faculty of Veterinary Sciences, Complutense University of Madrid, 28040 Madrid, Spain; irisazami@ucm.es (I.A.-C.); pauper21@ucm.es (P.P.-M.); 2 Department of Health Sciences, Faculty of Biomedical and Health Sciences, Universidad Europea de Madrid, Villaviciosa de Odón, 28670 Madrid, Spain; 3 Facultad de Veterinaria, Universidad Alfonso X el Sabio, Villanueva de la Cañada, 28691 Madrid, Spain; pmatamen@uax.es; 4 Department of Animal Health and Public Health, Catholic University of Valencia, 46002 Valencia, Spain; jose.sansano@ucv.es; 5 GREFA (Grupo de Rehabilitación de la Fauna Autóctona y su Hábitat), Monte del Pilar, 28220 Madrid, Spain; fgonzalez@grefa.org; 6 Departmental Section of Pharmacology and Toxicology, Faculty of Veterinary Science, University Complutense of Madrid, 28040 Madrid, Spain; 7 Department of Microbiology and Parasitology, Faculty of Pharmacy, Complutense University of Madrid, 28040 Madrid, Spain

**Keywords:** *L. infantum*, wild mammals, PCR, European hedgehog, red squirrel, European badger

## Abstract

Zoonotic leishmaniasis caused by *Leishmania infantum* is distributed worldwide and affects humans and domestic and wild mammals. In Europe, specifically in the Mediterranean basin, leishmaniasis is endemic due to the concurrence of the phlebotomine vectors and reservoir mammals, including carnivorous wildlife species and other less studied wild species. In this article, spleen, skin, and eye or oral swabs taken from 134 wild mammals admitted to five wildlife recovery centers in Spain were used. PCR employing fragments of the Repeat region, ITS1, and SSUrRNA were used for detection, and positive samples were processed for sequencing. *L. infantum* was detected in three out of the nine species analyzed, including European hedgehog, European badger, and red squirrel, with percentages ranging from 11.53 to 35.71%, depending on the species. Most of the species showed higher percentages of positivity in spleen samples than in skin samples. A small number of animals from the remaining six species tested negative, including Algerian hedgehog, stone marten, least weasel, garden dormouse, western polecat, and Egyptian mongoose. Hedgehogs and badgers are good candidates for consideration as epidemiological sentinels and pose a higher risk as potential reservoirs of leishmaniasis based on their percentage of infection and wide distribution.

## 1. Introduction

Leishmaniasis is a zoonotic disease caused by a protozoan parasite of the genus *Leishmania*. The Genus includes more than 20 zoonotic species with a worldwide distribution. *Leishmania infantum* is the most frequently reported species in the Mediterranean basin, causing a life threatening disease in humans and animals [1,2,3]. Cutaneous and visceral leishmaniosis (CL and VL, respectively) can occur [4]. In the first case, the promastigote stage of the parasite infects phagocytic cells from the skin and, eventually, the infected cell bursts, liberating amastigote forms to infect surrounding cells, while systemic disease (VL) occurs when infected cells and amastigotes spread via the blood to infect phagocytic cells in the bone marrow, liver, spleen, and lymph nodes [1]. The main route of transmission is the bite of a phlebotomine sandfly (order Diptera, family Psychodidae), though they are minor routes of infection, such as venereal or vertical transmission, which is proven in canids, or the oral route, which is described in hamsters and suggested in insectivorous bats [2,5,6]. About ten species of the genus *Phlebotomus* have been described as capable of transmitting *L. infantum* in southern Europe, with the most common being *P. perniciosus* and *P*. *ariasi*, which have a wide distribution in countries such as Italy, Portugal, France, and Spain [7,8]. Humans and domestic animals, such as dogs, have been the most widely studied reservoirs due to their high prevalence and relevance, but in the last few decades, several domestic and wildlife species have been analyzed to investigate their ability to maintain the biological cycle of the parasite [2,3,9]. The role of each host species in the spread of leishmaniosis in urban, peri-urban, and rural environments needs to be clarified. The first step should be the identification of potential hosts and, eventually, their ability to play a relevant role in outbreaks of this parasitosis, which can be proven through xenodiagnoses experiments. This approach was employed during the large outbreak of leishmaniosis in Madrid, when hares were proven to play a role in the maintenance of the cycle [10].

In Europe, wild carnivores, like foxes (*Vulpes vulpes*), jackals (*Canis aureus*), and wolves (*Canis lupus*), have been extensively studied due to their similarity to dogs, but other species, such as European hedgehog (*Erinaceus europaeus*), European badger (*Meles meles*), common bat (*Pipistrellus pipistrellus*), rodents (*Mus musculus*, *Rattus rattus*, etc.), red squirrel (*Sciurus vulgaris*), European and American minks [11,12] and martens (*Martes martes*), among others, have tested positive for *L. infantum* via both molecular and/or serological methods [2,3], although most of these studies employed few animals of each species. The growing interest in these species’ roles as potential pathogen reservoirs is caused by the increasing interaction between wildlife and urban and peri-urban environments due to the fragmentation of their habitats, the extension of peri-urban areas into typically wild territories, and the increase in eco-tourism activities. These situations led to increased interactions between wildlife, humans, domestic hosts, and vectors [9]. 

Regarding the diagnostic techniques employed for the detection of the parasite, serology is mainly used in humans and dogs, as it requires blood and is a minimally invasive procedure. However, in some of the wildlife species studied, serology was complemented with molecular techniques, with the most employed methods being PCR and qPCR [2]. Numerous targets of DNA have been described for use in the detection of *L. infantum*, with kDNA and SSUrRNA being the most commonly used due to their high sensitivity, but other targets, such as the Repeat region, also displayed high sensitivity, and the ITS1 target is preferred due to its higher specificity [10,11,13]. Blood, liver, and spleen are common samples used in DNA extraction and PCR, while in recent years, samples obtained via less invasive methods, such as hair and oral or ocular swabs, have been used, [2].

Considering that Spain is a country with high levels of prevalence of *L. infantum* in Europe and the Mediterranean area [14], the aim of this work was to analyze the occurrence of the parasite in wild mammals that are potential reservoirs of leishmaniosis in wild, urban, and peri-urban areas. For this purpose, different samples from the animals and several DNA targets were employed. The usefulness of less invasive techniques in testing wild species is evaluated in this work.

## 2. Materials and Methods

### 2.1. Origin of Animals and Samples

Samples were obtained, at convenience, from wildlife recovery centers in Spain. In total, 123 animals from 3 different mammal species were studied, including 83 European hedgehogs (*Erinaceus europaeus*), 26 red squirrels (*Sciurus vulgaris*), and 14 European badgers (*Meles meles*). The minimum number of animals included in each sample to detect the disease in these three species was calculated by employing previously described percentages of infection for each species: *n* = 11 for European badger (26% of infections) [15], *n* = 11 for red squirrel (25% of infections) [13], and *n* = 8 for European hedgehog (34.4% of infections) [13]. The online resource Working in Epidemiology (WinEpi) was employed to complete the calculation [16].

In addition, a small number (*n* = 11) of other six species were included in the study: Egyptian mongoose (*Herpestes ichneumon*), Algerian hedgehog (*Atelerix algirus),* stone marten (*Martes foina*), least weasel (*Mustela nivalis*), garden dormouse (*Elyomis quercinus*), and western polecat (*Mustela putorius*). 

The animals were admitted between November 2015 and February 2023 to five wildlife recovery centers: GREFA (Group of Rehabilitation of the Autochthonous Fauna and their Habitat; Madrid, Spain), “La Granja de El Saler” (Valencia, Spain), Mini-zoo (Guadalajara, Spain), “El Chaparrillo” (Ciudad Real, Spain), and CERI “Iberian birds of prey Studies Center” (Toledo, Spain). The areas of origin of the animals were the regions of Madrid, Castilla–La Mancha, Castilla y León, and the Valencian Community, Spain (Table S1). All animals were admitted to the recovery centers due to illness or trauma, and most of them (128/134) were roadkill or humanely euthanized following veterinary criteria and the AVMA guidelines for the euthanasia of animals [17]. Samples from skin, spleen, and/or liver were obtained from the dead animals and kept frozen until analysis. Six of the European hedgehogs recovered from their injuries and were reintroduced into the wild, but before that stage and during veterinary examination, oral and ocular swab samples were taken and kept frozen. In addition, oral and ocular swab samples were taken at the time of admission from two of the dead hedgehogs (Table 1).

### 2.2. DNA Extraction

DNA was isolated from the samples using 10 (spleen) or 20 mg (ear skin or liver) of tissue sample based on the kit manufacturer’s recommendations. The Thermo Scientific GeneJet Genomic DNA Purification kit (Thermo Fisher Scientific, MA, USA) was employed, and the manufacturer’s protocol was followed, except for DNA elution, which was carried out in 60 µL of elution buffer, instead of 100 µL. Sample derived from *L. infantum* promastigotes in culture and DNase free water were employed as positive and negative controls, respectively, in each batch.

### 2.3. PCR of L. infantum and Sequencing

DNA isolated from each sample was used in the detection of *L. infantum* using three different targets of DNA: Repeat region, SSUrRNA, and ITS1 [18,19,20]. The latter was only performed in samples positive at the other two targets, as its sensitivity was lower [11]. In the case of SSUrRNA and ITS1, a nested PCR (nPCR) was used. In all instances, the reaction was carried out in 25 µL, using 12.5 µL of the Supreme NZYTaq II (NZY Tech, Lisbon, Portugal) and 1 µL of each primer at 10 µM. For the Repeat Region and ITS1, 2.5 µL of DNA was used, and for the SSUrRNA, 5 µL of DNA was employed. The second reactions of both nPCRs were carried out using 5 µL of 1/40 dilution of the first PCR product or 1/20 dilution in case of faint positive reactions. For the Repeat region, primers T2 and B4 [20] were used; for SSUrRNA, primers R221 and R332 were employed for the outer section, and primers R223 and R333 were employed for the inner section [18]. Finally, for ITS1, outer primers LITSR and L5.8S and inner primers SAC and VAN2 were selected [19]. Moreover, positive (*L. infantum* promastigotes DNA) and negative (DNase free water) controls were employed in each reaction. For all PCR reactions, an initial step at 95 °C for 5 min was used to activate the enzyme, and a last step at 72 °C for 10 min was added for the elongation step. The intermediate steps, as well as other features, are described elsewhere [11]. Electrophoresis was carried out on 1.5% agarose gel stained with SYBR^®^ Safe DNA gel stain (Invitrogen, Carlsbad, CA, USA) and visualized under UV light. Positive samples displayed a band on the gel related to the expected size of each target, including positive controls.

### 2.4. DNA Sequencing and Alignments

All positive samples were cleaned and sequenced by a commercial company (Macrogen Spain, Madrid) using DNA Engine Tetrad 2 Peltier Thermal Cycler (BIO-RAD) and ABI BigDye^®^ Terminator v3.1 Cycle Sequencing Kit (Applied Biosystems, Waltham, MA, USA), following the manufacturer’s protocol. The obtained sequences were manually checked and aligned with Molecular Evolutionary Genetics Analysis (MEGA X) software and FinchTV 1.4.0 software (Geospiza, Inc.; Seattle, WA, USA). The consensus sequences of each positive sample were then compared with other available sequences using the BLAST tool (National Library of Medicine, Rockville, MD, USA) [21]. All obtained sequences of more than 200 base pairs were submitted to GenBank for accession number identification.

### 2.5. Phylogenetic Trees

Phylogenetic trees were constructed using sequences available from GenBank, together with sequences obtained in this study that were of sufficient quality and length. Only trees for SSUrRNA and ITS1 regions are shown, since there were enough sequences from other *Leishmania* species obtained from different authors available at GenBank. The Repeat region was only employed for detection and estimation of the percentage of homology.

Sequences of the ITS1 region had a minimum length of 290 nucleotides, and a sequence of *Trypanosoma cruzi* was included as an out-group reference. Sequences of the SSUrRNA region with a minimum length of 320 nucleotides were included, and a sequence of *Trypanosoma cruzi* was included as an out-group reference. In addition, sequences of *L. infantum* and other species of *Leishmania* derived from different sources were included to verify similarity. The evolutionary history was inferred using the Maximum Likelihood method based on the Tamura–Nei model [22]. The tree with the highest log likelihood was shown. The percentage of trees in which the associated taxa clustered together was shown next to the branches. Initial trees for the heuristic search were obtained automatically by applying the Neighbor-Joining and BioNJ algorithms to a pairwise distance matrix estimated using the Maximum Composite Likelihood (MCL) approach and selecting the topology with superior log likelihood value. The tree was drawn to scale, with branch lengths measured based on the number of substitutions per site. The analysis involved 26 nucleotide sequences. Codon positions included were 1st + 2nd + 3rd + Noncoding. All positions containing gaps and missing data were eliminated. Evolutionary analyses were conducted via MEGAX [23]. Bootstrap of 2000 replicates was employed to estimate the reliability of the trees.

### 2.6. Maps

The maps were created by taking as reference the map of prevalence of *Leishmania* spp. reported in wild animals [14] and the distribution of each animal species in Spain based on the atlas and red book of terrestrial mammals of Spain [24]. The AutoCAD program (Autodesk^®^ Inc., the Landmark, San Francisco, CA, USA) was employed to observe the overlaps between the two maps.

### 2.7. Statistical Analysis

A descriptive statistical analysis with absolute (n) and relative frequency of infection (%) was carried out, and a confidence level of 95% was estimated by employing the free online tool WinEpi (Working in Epidemiology) [16]. 

## 3. Results

### 3.1. Ocurrence of Leishmania spp. in Different Animal Species

In total, 19 of the 128 necropsied animals (14.84%, CI 8.68–21%) tested positive for *L. infantum* in at least one sample (spleen or skin) and one PCR target (Table 2 and Table S2). Positives were found in spleen or ear skin, while oral and ocular swabs (n = 8) were all negative. Only two animals tested positive in both spleen and skin: a breeding European hedgehog and an adult European badger (Table S2). The highest percentage of infection was found in European badgers (35.71%, CI 10.63–60.8%), followed by European hedgehogs (14.29%, CI 6.5–22.07%) and red squirrels (11.53%, CI 0.0–23.8%). 

### 3.2. Geographic Origin of the Samples and Risk of Infection

**Figure 1:**
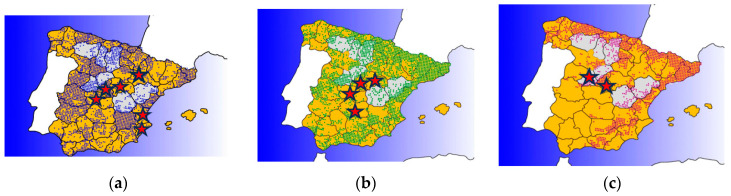
Map showing the distribution of species that tested positive for *L. infantum* in Spain (dotted areas). Areas colored in orange are recognized as having endemic leishmaniasis, according to ECDC, 2022. (**a**) Distribution areas of European hedgehog; (**b**) distribution areas of European badger; (**c**) distribution areas of red squirrel. Red stars indicate the origin of the samples used in the present study.

The area of leishmaniosis reported in humans and animals includes most of the Iberian Peninsula, which is in coincidence with the distribution of some of the species that tested positive in this study, namely European badger and European hedgehog, while red squirrel displayed a smaller area of distribution (Figure 1). The provinces in which animals were found belong to Madrid, Comunidad Valenciana (Valencia and Alicante), Castilla–La Mancha (Guadalajara, Toledo, Ciudad Real), and Castilla y León (Ávila), and their locations are marked with a star in the figure. Most of the animals were found in peri-urban areas, since many of them were roadkill, although some red squirrels and hedgehogs were also recovered from streets and urban gardens (Table S1).

### 3.3. Sensitivity of PCRs

A total of 253 samples were first analyzed at the Repeat region and the SSUrRNA fragment, and only positive samples obtained via these previous PCR protocols were tested at the ITS1 region (*n* = 20) (Table S2).

Considering all samples, the Repeat Region and the SSUrRNA PCRs displayed similar sensitivity, since 13 and 9 samples were positive, respectively. In total, 4.03% (5/124) and 7.08% (8/113) of positive samples were obtained in spleen and skin, respectively, when employing the Repeat region compared to 6.45% (8/124) and 0.89% (1/113) of positive samples rendered via the SSUrRNA PCR. The European badger displayed a higher number of positive samples when employing the SSUrRNA PCR of the spleen, while higher number of positives were obtained via the Repeat region PCR using the skin in European hedgehogs. The ITS1 tested positive in 4/12 and 1/9 samples in spleen and skin, respectively.

### 3.4. Sequences 

All samples positive to PCRs were sequenced and submitted to GenBank, although faint reactions did not render a legible sequence. The accession numbers are as follows: OP594494–OP594504 for sequences of the Repeat region; OP588913–OP588918 for sequences of the SSUrRNA; OP588937 and OP588938 for samples positive at ITS1 PCR. 

A total of 6 sequences of the SSU target, 11 of the Repeat region, and 2 of the ITS1 target were successfully aligned in both directions and subjected to BLAST analysis. The rest of PCR positive samples rendered short sequences, since the signal was low. All of the samples corresponded to *L. infantum*, with percentages of homology being 99–100%.

### 3.5. Phylogenetic Trees 

**Figure 2:**
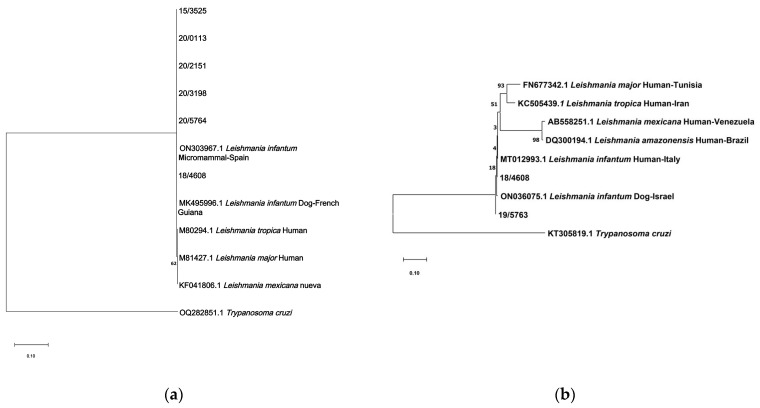
Phylogenetic trees of SSUrRNA (**a**) and ITS1 (**b**) fragments. The trees were constructed by applying Neighbor-Joining and BioNJ algorithms to a matrix of pairwise distances estimated using the Tamura–Nei model and selecting the superior log likelihood value. The trees are drawn to scale, with branch lengths measured based on the number of substitutions per site. A bootstrap of 2000 replicates was employed.

Phylogenetic trees were constructed by employing sequences from the two selected targets after cutting the sequences of the primers employed via the PCRs (Figure 2). The Repeat region was excluded since it had very short sequences. All sequences obtained for SSUrRNA had 320 bp and were identical, since it is a conserved region. These sequences were also identical to reference sequences from GenBank, a sequence from a dog derived in French Guiana (acc. number MK495996), and a sequence from a micromammal derived in Spain (acc. number ON303967).

The two sequences of the ITS region were employed to construct the phylogenetic tree with 290 bp. Although ITS1 sequences were grouped very close together in the phylogenetic tree, a slight difference was found between both sequences in this study. One sequence obtained from the spleen of a European hedgehog (18/4608) was closer to a *L. infantum* sequence derived from a human in Italy, while the other sequence obtained from the skin of a European hedgehog (19/5763) was aligned in a slightly more distant position. 

## 4. Discussion

In this study, a high number of samples derived from wild animals were analyzed to determine the presence of *L. infantum*. Most of them had already been studied previously for the presence of *L. infantum*, but either a different target (kDNA) was employed in some of the studies [25] or a small number of samples or animals were included. In the present study, the most relevant studied species are the red squirrel, the European badger, and the European hedgehog, given the number of animals from each species that tested positive and their wide distribution in Spain and other Mediterranean countries.

The results obtained for the European badger, with 35.71% of animals testing positive, are in line with other studies conducted in endemic or peri-endemic areas of Spain and Italy, where results ranged from 26 to 53.3%. In both cases, spleen samples were analyzed (and, in some cases, liver), with different DNA targets, ITS2, and kDNA [15,25]. This diversity of targets may influence the results, as not all targets are equally sensitive. In our case, positive results were obtained via SSUrRNA nPCR, as a target had high sensitivity when a nested PCR was employed, as was demonstrated in other articles with other wildlife species [11,18,19,26]. In the present study, a substantial percentage of the animals were found to be infected, all of which were roadkill animals from peri-urban environments. Badgers are long-living animals, and for that reason, their role as potential reservoirs of the disease is relevant [24].

In the case of hedgehogs, most studies focused on species of the genera *Atelerix* and *Paraechinus* [27,28,29], with only two studies working with samples from *Erinaceus europaeus* [13,30]. In our case, the latter species is the one that tested positive for *L. infantum*, with 14.29% of samples being positive at the spleen or ear skin. These results are slightly lower than those obtained in the studies carried out by Muñoz-Madrid et al. [30] (100% positive results in hedgehog hair, albeit using only one specimen) or Alcover et al. [13] (34.4% positive spleen and skin samples). In both cases, the target used for *L. infantum* detection was kDNA, which could have influenced this difference in results. kDNA is one of the most widely used targets in the detection of *L. infantum*, albeit with highly variable results, ranging from studies with no positive samples to studies with remarkably high percentages of positivity [11,13,31]. The animals analyzed in the present study came from peri-urban areas, and it is common to see them near human settlements. They inhabit nests, and these habitats could be protected places in which phlebotomine sandflies live, as is often the case in other small mammals’ nests [24].

Red squirrels have scarcely been investigated for the presence of *L. infantum*, except for one study conducted in Catalonia, in which the authors found 25% of the animals to be infected [13]. In our study, 11.53% of red squirrels were positive, i.e., a slightly lower result, but this result could also be attributed to the origin of the animals. The analyzed specimens in this study were from urban or peri-urban areas, and they inhabited gardens and parks with trees, which can be considered as potential risk areas in case leishmaniosis outbreaks occur, as they are suitable habitats for the reproduction and maintenance of sandflies [24].

In the present study, several targets were used for the detection of *L. infantum* infection: the Repeat region and a fragment of the SSUrRNA. SSUrRNA displayed high sensitivity, according to previous studies, but better results were observed in hedgehogs and red squirrels employing the Repeat region, and this latter finding agrees with previous studies that employed this last target and obtained high percentages of positivity in common urban bats and American minks [11,32]. Other authors who employed the Repeat region found higher numbers of positive results compared to other DNA targets, such as kDNA, Mini-exon or SSUrRNA [29,33]. Authors such as Lauchaud et al. [33] have described a high degree of inhibition when using the Repeat region as a target for the detection of *L. infantum* in humans, albeit with variable results in other animal species. It appears that the Repeat region works differently for distinct animal species, a fact also observed in the present study.

Regarding the results obtained via ITS1, we found a small number of positive results, given that we only used this target in those samples that were positive for other DNA sites. There are numerous scientific articles that discuss the detection of *L. infantum* with ITS1, and the authors always highlight the high specificity of this region, but it has a lower level of sensitivity [19,34,35]. Indeed, this was the case for a previous report of *Leishmania* in hedgehogs [29]. Therefore, it is particularly useful when sequencing and determining the species or genetic variants of *Leishmania* present in the infected animals, but it has the disadvantage of lower sensitivity. In our study, we only obtained five positive samples for this target, despite performing a nested PCR to increase the sensitivity.

The results obtained in this study showed a higher percentage of positives using spleen samples (*n* = 12/124) compared to other tissue samples, such as skin (*n* = 9/113) (9.52% vs. 7.96%) (Table S2). This result agrees with other studies, in which this organ was the preferred site (the most sensitive target) for the detection of *Leishmania* [32,34,36,37]. However, it is necessary to handle this type of sample with care, as it can have a high number of inhibitory enzymes and proteins, according to DNA extraction protocols, and this situation can lead to false negative results. In this study, DNA extractions of these samples were performed using a lower amount of tissue (10 mg) than other samples, as indicated in the manufacturer’s instructions. In any case, the spleen, as well as other animal tissues, such as the lymph nodes or bone marrow, accumulate high numbers of parasites, which have a high tropism toward these areas, where they easily multiply [38].

Another tissue sample frequently used for the detection of *L. infantum* in this study and other studies is the skin, but the results are highly variable. This issue could be due to the difficulty of detecting these parasites in the absence of lesions. In the case of the animals studied here, none presented lesions compatible with *L. infantum*, which might be one reason that *L. infantum* is detected in skin samples less frequently than in the spleen. However, this observation was not true in hedgehogs, which displayed a higher percentage of positivity in skin (10.93% vs. 6.67%). Another explanation is that the distribution of the parasite depends on the analyzed species. Our findings agree with other studies, in which hedgehogs show similar or even higher percentages of positivity in skin than in spleen samples [28,39]. 

It would be interesting to analyze a larger number of less invasive samples, such as eye and oral swabs, which, in this study, have not yielded positive results, but encouraging results can be found in the literature, mainly in dogs and hedgehogs [27,38]. The drawback would be that samples are difficult to obtain, as experienced handling is needed to obtain a good sample capable of yielding conclusive results. Another reason that explained the negative results of these samples is that the animals may not have a large number of parasites, and only one of the two animals analyzed using skin and spleen tested positive in spleen. Again, no lesions were observed in the oral or ocular mucosae, and many of the animals (6/8) were released in the wild. 

Considering the overlapping distribution of wild species positive for *L. infantum* infection with the distribution of the parasite in the Iberian Peninsula, we considered that the species with the highest risk of being potential reservoirs are badgers and hedgehogs. Also, we would like to highlight that the information provided in this article is relevant to developing conservation strategies and management of wildlife populations. Finally, the research of zoonotic diseases in wildlife species make them sentinels that can be used to explore the epidemiological situation of endemic or emerging diseases.

## 5. Conclusions

The present study analyzed the occurrence of *L. infantum* in nine wild species of mammals, three of which tested positive for the parasite. The European badger was the species with the highest percentage of infection (35.71%). Lower percentages were found in red squirrel (11.53%) and European hedgehog (14.29%). Considering the distribution of the wild species analyzed and the parasite in the Iberian Peninsula, European hedgehogs and European badger could pose a higher risk of being potential reservoirs of leishmaniasis. Wild animals could act as sentinels to evaluate the epidemic status and predict emerging situations. 

## Figures and Tables

**Table 1:** Wild animal species analyzed for the presence of *L. infantum*. Numbers of animals and types of samples are included.

Scientific Name	Number of Animals (*n*)	Type and Number of Samples			
		Ear Skin	Spleen	Ocular Swab	Oral Swab
*Erinaceus europaeus*	83	64	75	8	8
*Sciurus vulgaris*	26	25	24 *	-	-
*Meles meles*	14	14	14 *	-	-
*Atelerix algirus*	3	3	3	-	-
*Martes foina*	2	2	2	-	-
*Mustela nivalis*	2	2	2	-	-
*Eliomys quercinus*	1	1	1	-	-
*Mustela putorius*	1	-	1	-	-
*Herpestes ichneumon*	2	2	2	-	-
TOTAL	134	113	124	8	8

* One animal analyzed using sample of liver tissue, instead of spleen, due to extensive damage caused to the spleen.

**Table 2:** Animal species and samples evaluated for *L. infantum* by employing different PCR targets. Number of positive samples and percentage of positivity is shown.

Species	Spleen (+/*n*)			Ear Skin (+/n)			TOTAL (% Value, 95% CI)
	Repeat Region	SSUrRNA	ITS1	Repeat Region	SSUrRNA	ITS1	
*Erinaceus europaeus*	2/75	3/75	1/5	6/64	1/64	1/7	11/77 (14.29%, CI 6.5–22.07%)
*Sciurus vulgaris*	3/24	1/24	0/3	0/25	0/25	N.A.	3/26 (11.53%, CI 0–23.8%)
*Meles meles*	0/14	4/14	3/4	2/14	0/14	0/2	5/14 (35.71%, CI 10.63–60.8%)
*Atelerix algirus*	0/3	0/3	N.A.	0/3	0/3	N.A.	0/3 (0%, CI 0–63.16%)
*Mustela nivalis*	0/2	0/2	N.A.	0/2	0/2	N.A.	0/2 (0%, CI 0–77.64%)
*Martes foina*	0/2	0/2	N.A.	0/2	0/2	N.A.	0/2 (0%, CI 0–77.64%)
*Eliomys quercinus*	0/1	0/1	N.A.	0/1	0/1	N.A.	0/1 (0%, CI 0.0–95%)
*Mustela putorius*	0/1	0/1	N.A.	N.A.	N.A.	N.A.	0/1 (0%, CI 0.0–95%)
*Herpestes ichneumon*	0/2	0/2	N.A.	0/2	0/2	N.A.	0/2 (0%, CI 0.0–77.64%)
TOTAL	5/124	8/124	4/12	8/113	1/113	1/9	19/128 (14.84%, CI 8.68–21%)

n: number of animals. N.A.: not analyzed. Eight oral and ocular swabs from European hedgehog were negative for *L. infantum* PCRs and are not included in the table.

## Data Availability

Sequences obtained in this study were deposited at GenBank with the following accession numbers: OP594494–OP594504 for sequences of the Repeat region; OP588913–OP588918 for sequences of the SSUrRNA; OP588937 and OP588938 for samples positive at ITS1 PCR.

## Supplementary Materials

The following supporting information can be downloaded via the following link: https://www.mdpi.com/article/10.3390/pathogens12081048/s1, Table S1: Data of the animals employed in this study; Table S2: PCR results by animal sample and target.
